# Creating Parent Capacity in Cases of Selective Mutism

**DOI:** 10.3390/bs16010113

**Published:** 2026-01-14

**Authors:** Heidi Omdal

**Affiliations:** Department of Education, University of Agder, Universitetsveien 25, 4630 Kristiansand, Norway; heidi.omdal@uia.no

**Keywords:** capacity-building, parent guidance group, selective mutism

## Abstract

This paper draws on a capacity-building initiative by the researcher to prepare the parents of nine selectively mute (SM) children to take the lead in their child’s change process towards, gradually and in tiny steps, starting to speak in more situations and to more people. Egan’s skilled helper model is used as a framework in the parent guidance group. Focus group interviews in the parent group make up the data set for the project, along with the parents’ written answers to questions arising from Egan’s skilled helper model between meetings. Content analysis is used to analyse the data. A common theme among the families is how to find the right balance between supporting and challenging the SM child in communication with others. How to promote greater independence in parent–child relationships is the main question from the project.

## 1. Introduction

Children with selective mutism (SM) constantly avoid speaking in specific social situations. Often, they remain silent in school and kindergarten, while speaking normally to parents and siblings at home (American Psychiatric Association [APA], 2022). SM is understood as a specific phobia of expressive speech (Omdal & Galloway, 2008), rather than a symptom of social phobia (Kristensen, 2002). Previous research found that parents of SM children were more over-protective and controlling than parents of children with other anxiety disorders or control children without anxiety conditions (Alyanak et al., 2013; Edison et al., 2011). Parents of SM children report lower levels of authoritative parenting than parents of children without any disorders (Slobodin et al., 2025). So, parents tend to overprotect the SM child or permit withdrawal from joint activities when the child communicates fear, is reluctant to try something new, or refuses to engage with other children (Omdal, 2014; Snyder et al., 2013). Thus, this group of children may have few coping experiences in their environment, and their self-efficacy may become diminished (Bandura, 1997; Omdal & Galloway, 2008). When no one communicates an expectation that the child will be able to master a task, the child will mirror themself in this negative feedback from their environment, lose faith in their ability to master the task, and stop trying (Bandura, 1997; Mead et al., 2015). Early intervention (Eng et al., 2017) and close cooperation with parents are crucial to prevent serious mental health problems in children with selective mutism (Oerbeck et al., 2014; Omdal, 2008). Earlier research has shown that parenting interventions that teach parents how to maximize the child’s opportunities to speak while eliminating accommodation strategies reduce SM symptoms (Shorer et al., 2023).

This paper considers an innovation process in a parent guidance group with nine SM families phasing in an authoritative parenting style (Baumrind, 1991; Snyder et al., 2013; Wentzel, 2002) in interactions with their SM child. An innovation is a planned change intending to improve practice (Skogen, 2004). Authoritative adults (Baumrind, 1991; Snyder et al., 2013) try to find the right balance between, on the one hand, challenging the child, setting limits, and making demands and, on the other, offering support, being genuinely interested in the child’s perspective, and building a warm, trustful relationship with the child by spending time with them based on their interests. An authoritative parenting style differs from three other adult styles illustrated along the two axes of warmth and control; authoritarian (low warmth and high control), permissive (high warmth and low control) or neglecting (low warmth and low control) (Baumrind, 1991). Baumrind (1991) found that the authoritative parenting style, characterised by high warmth and high control, promoted positive behaviour and decreased negative behaviour in children. Positive interactions between parents and children are crucial for children’s early learning and development (Downer et al., 2009).

In cases of SM, capacity building is required to move from silence to speech, and parents are viewed as crucial change agents for the SM child. At the same time, without any guidance from professionals with SM competence, parents are potential risk factors for the child’s progress (Omdal, 2008). When children constantly avoid speaking, early intervention is urgent to prevent deadlocked communication patterns between parents and children and negative expectations towards the children’s coping possibilities in their environment. The parents in this study received guidance throughout the process, based on the reflection questions in Egan’s skilled helper model (Egan, 2014). This skilled helper model thus created a clear common framework around the parent group, making it easier to translate the core principles of the project into practice (Blase et al., 2012; Durlak & DuPre, 2008; Fullan, 2016; Greenberg et al., 2005).

The researcher started a parent guidance group with nine SM families in 2017, holding six meetings, or assemblies, where parents exchanged experiences and received guidance from the researcher. The researcher was also the facilitator as it was seen as crucial with long experience and knowledge on SM to carry out this intervention. Very few have this capacity in Norway. Each assembly was scheduled with three hours with the facilitator present. Parents could stay together longer if they wanted to. A shared meal was included in the three hours. We sat around a table during the assemblies, making it possible to see each other and listen very carefully. It took place in the middle of the day on Saturdays due to parents’ working life and long distance to drive to the University where it took place. The parents were supposed to prepare for the second, fourth and fifth assembly by answering questions they were e-mailed from step 1–3 in Egan’s skilled helper model. These steps formed the structure for the assemblies. The first assembly was carried out in February, the second in March, the third in April, the fourth in September, the fifth in October and the sixth and last one in November. Before we met, the parents received an information letter about the project with a consent declaration they had to sign if they were willing to participate in anonymous focus-group interviews at the end of the project year. They obtained written information about Egan’s skilled helper model, so they knew the structure of the assemblies. The information made it clear that success depended on their active contribution in supervision and acting towards the goals with their child. On the first assembly, we tried to get to know each other. All nine families were present and informed each other about the situation with the SM child. The facilitator made notes which were sent back to them to check if we received it right. On the other assemblies, there were four to five families present, and it varied which families. The facilitator seldom got answers to e-mail reminders about the assemblies, so it was impossible to know how many would come. One family was always there, as they had agreed with the helping services to be there as part of their treatment. On the second assembly, we reflected more on the current situation. The participants had received an e-mail with some questions to reflect on according to Egan’s (2014) step one in the skilled helper model. Four families answered the questions and sent them in an attachment to their e-mail response which made it easier for the facilitator to prepare for the assembly. On the third assembly, the participants were invited to join a course on SM with friends, family and professionals around the child. One family brought with them their child’s special needs teacher, otherwise there were just the child’s parents. We focused on the parent role as an authoritative adult towards the SM child’s special needs. The fourth assembly took place after there had been arranged two weekend assemblies, one in May and one in August, for the whole families with low pressure activities which the children could master. It gave the facilitator a picture of challenges and possibilities in each case. Before the fourth assembly, the participants were supposed to answer questions in an e-mail about the preferred situation to prepare themselves and the facilitator for making goals when they met. Five families sent answers. The fifth assembly was about acting strategies. Two families sent their answers to questions around this. They were supposed to reflect on an action plan when getting together. On the sixth assembly, evaluation of the project year and development was on the agenda.

The research project aimed to detect how parents of children with SM understand and act and to identify what might be hindering them from acting differently (Crick & Dodge, 1994) to remove any reinforcers for the child’s behaviour. The parents experienced several challenges with their SM child which they aimed to reduce or improve through the project. Overall, the problematic behaviors involved avoidance and withdrawal in different situations which the child perceived as frightening. The children needed to be in smaller groups if they were supposed to take the initiative in play and interactions with other children. At least four of the children found it easier to speak to strangers who did not know about their SM or had any expectations towards them. They could not inform others about their needs or ask for help if they could not speak to the person. Some communicated very little with their body language and showed limited facial expressions. One child did not tolerate loud sounds and noise which made it difficult in various situations at school, for instance in the gymnastics. Another child did not manage to be in the classroom without his mother by his side. A couple of children were sensitive about getting critique, so they would not write or do assignments at school. One child could not go to the toilet when outside home. Most of the children did not manage to be in a situation where they became the centre of others’ attention. At least three of the children did not dare to take the bus with peers or buy anything on their own in the shop. Two children did not dare to play in their friends’ house without their parents present. Parents felt that they had to take very small steps forward to avoid overwhelming their child, and that they had to educate others to do the same to avoid putting too much pressure on speech. The capacity building initiative in a parent guidance group resulted in revitalisation of the Norwegian SM Parent Support Association.

This study drew on empirical evidence from parents responsible for their SM child’s progress at home and in their spare time. The study questioned whether Egan’s skilled helper model (Egan, 2014) was adequate in phasing in the evidence-based key principles of the innovation outlined below.

### 1.1. The Parent Guidance Group

#### 1.1.1. Principles

The key principles underlying the capacity building in the parent guidance group are: (1) Authoritative parents build quality relationships with SM children, set standards in interactions within and outside the home, and strike the right balance between expectations/demands and support towards the SM child’s social communication. Authoritative parents (Baumrind, 1991) approach children with warmth, tolerance, and openness in their relationship, while establishing interpersonal values, norms, and standards in social interactions. Authoritative parents prevent the child’s over-dependency on themselves and aim to stay in the zone of proximal development with the child (Vygotskij et al., 1978). Through scaffolding, parents try to move the child from the actual level of development to the next potential level of development. As the child masters the task, the scaffolding is removed, enabling the child to complete the task again on their own. (2) Consistency is achieved when parents commit to shared visions, attitudes, and practices that underpin the innovation. Consistency is promoted among parents who pursue common learning measures and exhibit joint attitudes and actions through collective orientation. Egan’s (2014) skilled helper model provides the structure and goals of each parent assembly during the implementation of the key principles. (3) Continuity is key to the success of capacity building. Parents in the parent guidance group commit to remaining loyal, and to work systematically with the two other principles (mentioned above) to ensure long-term effects and sustainable development in the SM child. When implementing new actions as part of the innovation, parents should find ways of integrating these with former actions to continue the capacity building over time.

The parent guidance group works on developing and implementing the three key principles during a 12-month period, with the goal of instilling in the parents a mutual understanding and a shared loyalty towards the vision of an authoritative parenting style. During the 1-year programme, the parents are required to make goal-specific, time-scheduled plans, for the implementation of new activities and the organisation of interpersonal interactions with the SM child both within and outside the home environment. Parents are to promote the key principles to family members and friends to obtain a common evidence-based understanding of SM and consistent reactions towards the child in various contexts.

The key principles are grounded in SM theory (Alyanak et al., 2013; Edison et al., 2011; Oerbeck et al., 2014; Omdal & Galloway, 2008; Omdal, 2014, 2016; Slobodin et al., 2025), theory on authoritative parenting (Baumrind, 1991), symbolic interactionism (Mead et al., 2015), bioecological theory (Bronfenbrenner, 2005), social learning theory (Bandura, 1997) and implementation theory (Blase et al., 2012; Domitrovich et al., 2008, 2012; Durlak & DuPre, 2008; Fixsen et al., 2005; Fullan, 2016; Greenberg et al., 2005).

#### 1.1.2. Content

During the 1-year implementation phase, SM parents participate in six 1-day parent assemblies that cover the core components of the intervention:

(1) Selective mutism and its causes and intervention (Omdal, 2014, 2016), where information is provided on the nature and causes of selective mutism and how and why the intervention is carried out;
(2) The authoritative parenting style and development of quality relationships between children and adults (Baumrind, 1991; LaParo et al., 2012), which deals with the features of authoritative parenting and why this style is preferred in SM cases;
(3) Graded in vivo flooding (Omdal & Galloway, 2008), a technique emphasising approaching speech in small and gradual steps in cooperation with their parents. When a child overcomes its fear, with support from others in a safe environment, this can lead to coping experiences and increased belief that trying new behaviour in this environment works;
(4) Other people’s expectations towards the SM child, which addresses how expectations have the strongest impact on the child either withholding speech or recovering from the speech phobia (Mead et al., 2015; Omdal, 2008). The presupposition is that the child’s self-belief will be strengthened if a positive outcome of the implementation plan is expected and the child is encouraged to allow others to help them out of the anxiety. The child will benefit from those around them keeping to decisions made in cooperation with the child about trying to move forward, rather than withdrawing the child from exposure to frightening situations. This will help the child develop coping mechanisms and strengthen their motivation to make progress, extend speech, and participate in natural social interaction in their spare time;
(5) Implementation and capacity building, where it is emphasised that the parents, in each SM case, carry the main responsibility for ensuring that the implementation plan is followed in the child’s daily interactions within and outside the family. The parent guidance group represents a crucial driving force during the implementation phase, as the participants exchange knowledge and experiences gained during this phase. The parents share a common vision of extending their children’s communication and lead the work towards the vision, preparing the implementation plan with clear goals and measures in agreement with the facilitator/researcher in the parent guidance group. The researcher and facilitator in the parent guidance group give the parents the knowledge needed to support the implementation process in each SM case. Thus, the facilitator is an important driving force in the innovation (Fixsen et al., 2005). The researcher has developed a support system for participants, which includes an evidence-based book about SM (Omdal, 2016), covering the core components of the innovation. Participants are also given a summary of Egan’s (2014) skilled helper model, which shows them the structure and goals of each parent assembly during the 1-year implementation period;
(6) Evaluation and the way forward in each SM case, which is the last parent assembly, where the parents evaluate the SM child’s and their own progress over the implementation year and consider what it meant to them to be part of a guidance group with other SM parents.

### 1.2. Egan’s Skilled Helper Model as Framework for the Parent Guidance Group

**Figure 1:**
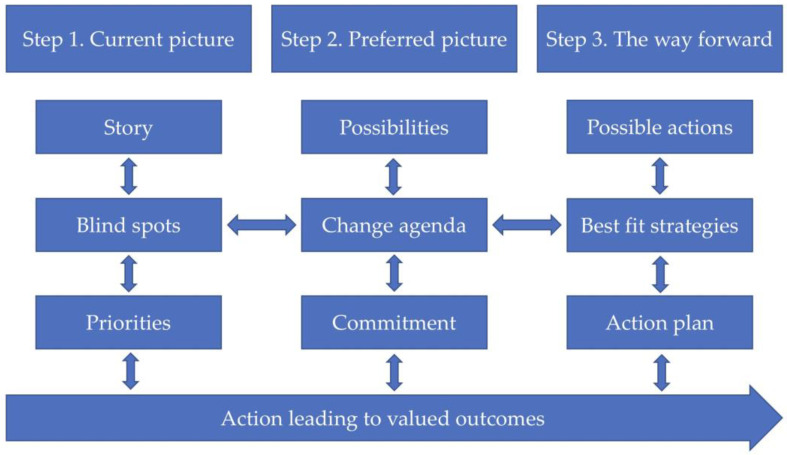
Steps in Egan’s skilled helper model (Egan, 2014).

Egan’s skilled helper model, which is used as an informed and agreed structure with the participants through the implementation year, is organised into three steps (see Figure 1): (1) the current picture, (2) the preferred picture, and (3) the way forward. The skilled helper model is flexible; the three steps are interrelated, and it is possible to move back and forth, and up and down in-between the part processes and steps during the change process according to the participants’ changing needs, challenges, goals, and strategies at any time. The participants are expected to be active during the entire process of implementing goals, decisions, and actions towards a changed situation. Continuous evaluation throughout the whole change process is necessary to reach valued outcomes.

In step 1 containing story, blind spots and priorities, the participants identify their current situation with the child here and now. The parents tell their story in the group. With help from the group and facilitator they try to identify the most challenging situations for their child and family. What is going on in these situations? Are there any unused opportunities or blind spots, something unconscious or hidden for oneself, in making these situations change? What hinders parents in implementing the change? What do the parents feel that they need from others to make change happen? What will they prioritize in their daily life to make it easier for the SM child? The parents identify their own needs to become motivated to implement the change process.

In step 2 containing possibilities, change agenda and commitment, the participants identify their preferred situation with the child. What are the parents’ wishes, goals, and possibilities in making change happen? How would the family situation and spare time look if the child started to speak? Would all problems be resolved, or is there anything else that should be changed? Parents make a change agenda with clear goals for the child and themselves. The parents try to imagine a better future and gain new hope. They choose realistic, specific, and challenging goals intended to make change happen in the current situation. What do the parents prioritise among available solutions? What will they have to sacrifice to commit to staying loyal to the chosen goals and actions? What should the schedule look like?

In step 3 containing possible actions, best-fit strategies and action plan, the participants identify action strategies with possible constructive consequences in the way forward. The parents define concrete problem-solving actions or measures from their goals. What should they do to get what they need or want? What does the implementation plan look like? The core issues are defined in terms of concrete actions, making them possible to implement in practice. How to evaluate the strategies along the way to ensure that they work in the right direction is crucial to plan. What signs will they look for? The actions must be meaningful to the participants and fit their abilities (best-fit strategies), preconditions, available resources, personality, and schedule to ensure coping experiences. The parents receive help from the group and facilitator to create the implementation plan or the action plan, which is intended to help them to work systematically towards the strategic goals, eventually leading to the valued outcomes. As Egan’s skilled helper model is based on active participation and action in all steps from the one who seeks advice in order to succeed in making change happen, this is highlighted with the heading action leading to valued outcomes in the bottom of the figure.

During the last parent assembly, evaluation is the main goal. The participants are asked to identify what they feel that they have achieved from their identified wishes and needs, what they should continue to work on, and what has either helped or hindered the fulfilment of their implementation plan. They receive feedback on their efforts and are encouraged to continue working on the change process after the implementation period is finished.

### 1.3. Building Parent Capacity Through a Collective Orientation

To bring about change in parents’ strategies, all actors in the parent guidance group should move in the same direction and learn from each other through the exchange of ideas, experiences, and knowledge in a joint capacity-building process (Fullan, 2016). Fullan (2016) emphasises that a strong collective orientation strengthens participants’ motivation for change. Thus, an effective collaborative culture among parents might increase capacity (Fullan, 2016; Hargreaves et al., 2018).

In parent groups, a collective collaborative culture and capacity building might be nurtured by enhancing social interactions and strengthening shared learning processes and networking (Hargreaves & Fullan, 2012). Successful collaboration among parents in a similar situation with a child who does not speak depends on organising structured time together in which common learning processes might take place (Leithwood, 2019).

With these ideas in mind, Egan’s (2014) skilled helper model and motivational interviewing (Miller & Rollnick, 2013) were used as a way of articulating tacit knowledge in the parent group. Furthermore, a methodology called IGP (individual–group–plenary) was designed to stimulate collective reflective processes (Roland & Ertesvåg, 2018).

## 2. Materials and Methods

This paper is based on the results gathered by the author during the first year of implementation. The results were gathered from: (1) focus group interviews with the parents responsible for creating the capacity in each SM case in the parent guidance group; and (2) participating parents’ written answers to questions arising from Egan’s (2014) skilled helper model between parent assemblies.

Participants gave their informed consent to take part in the research, and the project was approved by the Norwegian Center for Research Data and followed the Guidelines for Research Ethics in the Social Sciences and the Humanities given by the National Committee for Research Ethics in the Social Sciences and the Humanities (NESH, 2023). The families were recruited through a Facebook group for parents with children with SM in Norway. The only criterion for participation was to have a child with SM. Nine families wanted to take part in the research. Eight of the SM children were between seven and twelve years old, attending ordinary, public schools. In one case, a six-year-old child was still in kindergarten. All except one child was diagnosed with SM by the helping services in preschool age. They did not suspect any other diagnosis in these children. The undiagnosed child did not have a diagnosis as the helping services found it hard to assess him when he could not speak. Though, they suspected autism. The diagnosis was confirmed for all children. All children had siblings without SM. All except one child lived together with both parents. They first noticed SM in kindergarten. All except the undiagnosed child had a special needs teacher following up the child in school.

Egan’s skilled helper model (Egan, 2014), which was used as framework for the project, claims that those seeking guidance should take an active role in the change process to become more motivated to follow the plan and implement goals and measures. The skilled helper model was based on the hypothesis that the expert advice of the facilitator would not be helpful until the advice-seeker had identified possible maintaining factors that perpetuated the silence in their specific case (Hong et al., 2013; Nordahl & Hansen, 2012). The reflection questions used in between the assemblies were intended to raise parent awareness. These were meant to help the parents articulate their thoughts and feelings in the specific situation, reflect on their needs, and arrive at appropriate courses of action. The parents’ answers to reflection questions arising from Egan at step 1–3 are reported in the results. Egan (2014) was assumed helpful in implementing authoritative parenting (Baumrind, 1991), as both theories expect clients to take an active role in their change process. Both approaches presuppose reflections on the current situation and the child’s special needs in social interactions before making goals and measures which ensure the balance between support and exposure to situations perceived by the child as scary. In other words, step 1–3 in Egan structured the implementation of the authoritative parenting in this project.

Content analysis (Miles et al., 2014; Patton, 2002) is used to analyse the data. Common themes across cases within the three steps they were asked to report from in Egan’s skilled helper model are analysed in NVivo (Richards, 2002) and we search for the participants’ “real world experiences” (Denzin & Lincoln, 2018) as objectively as possible. Our goal is to interpret the interviewees’ actions and social world from their own perspectives (Brinkmann & Kvale, 2015; Bryman, 2016).

## 3. Results

In the following, results from the parents’ written answers to questions arising from Egan’s skilled helper model between parent assemblies and from the focus group interview involving evaluation of the implementation year are presented. Quotes are in italics.

### 3.1. The Steps in Egan’s Skilled Helper Model

#### 3.1.1. The Current Picture

The parents based their description of the here-and-now situation with their child on questions derived from Egan (2014) the first time they met in the parent group. The topics addressed included particular challenges for the family owing to the child having SM, aspects the child struggled with the most, various positive situations, ways to do more of what was experienced positively, considerations to be made by others in order to achieve a good interaction with the child, and potential positive resources in the parents’ surroundings that could be capitalised on to support the child. The parents and facilitator tried to identify some key areas that could be changed in each case. Attempts were made to identify particularly challenging situations for the child and the family. Untapped opportunities for change and any blind spots or hidden factors preventing the parents from acting in a change-oriented manner were also explored.

The parents highlighted difficult situations, such as when they had to speak for their child when the child was addressed by others: “*The difficult situations are when strangers ask direct questions, and we have to speak for her*” (mother of a seven-year-old girl). Several mentioned that the child occasionally spoke to strangers who did not know that she was mute. These were experienced as good situations that provided the child with an experience of mastery: “*Good situations are when she dares to talk to strangers. In these situations, she does not think that the stranger knows about her mutism. The sense of achievement she gets in these situations makes her proud and happy, which is clearly visible in her*” (mother of a seven-year-old girl). The parents also described particular problems at school, where the child might avoid going to the bathroom, eating, or performing tasks: “*School and the interaction with classmates and teachers is challenging. She cannot communicate with teachers and is unable to play with more than one person at a time. She cannot accept any form of criticism at school. She struggles to go to the bathroom at school. What happens when these situations arise is that she locks herself up, refuses to work at school, does not eat at school, and becomes sad. She becomes frustrated, both with herself and with those around her. She often feels blocked during the lesson. She says it is difficult to relax her muscles and body. When the teachers approach her, her body becomes stiff”* (parents of an eleven-year-old girl). The parents emphasised that others should be patient, relaxed, and relate naturally to the child, without having excessive expectations for speaking, when trying to have a good interaction with the child. They were concerned that the child must feel safe in the environment and not be pressed. Concerning what the parents themselves could do to utilise the resources in their surroundings in order to ease the situation, they considered more connection of the SM child with other children and greater activation outside the home. They also planned to set small goals together with the child where the child needed to be challenged. One family wanted a support person for the child. The parents said they were trying to be clearer to others about the challenges of having a child with selective mutism.

#### 3.1.2. The Preferred Picture

The parents had the most input on questions regarding the desired situation. They had a clear desire and need for change. Five of the families thought through the questions related to the desired situation in advance and responded in writing via email before the parent meeting. The parents and facilitator were goal-oriented and opportunity-oriented. How would the family’s situation be if their child spoke with everyone? Would the challenges then be resolved, or was there more that needed to change than just increased speech? The aim was to envision a better future and develop a sense of hope. Each parent was to select some realistic, specific, and challenging goals to approach within a certain time frame. They needed to be aware of existing opportunities, priorities that would be required in order to make the necessary commitments, and the costs these commitments would entail. The parents believed that the situation for the child and the family would be much easier if the child could speak freely with everyone. According to the parents, this would make the child more independent and able to, for example, shop at the store by themselves, take the bus, visit friends, and give messages or ask others about something. The family could spend more time with others, without having to consider that their child would then have to avoid speaking for long periods of time.

The parents struggled mainly with setting goals for their child and themselves in the change process and with creating a timeline for how to observe progress in the child. Research shows that systematic work with increasing exposure to the child’s fears is a success criterion for overcoming the phobia of speaking (Omdal & Galloway, 2008). The parents discussed how they gradually tried over time to help their child reach new goals and try new things by encouraging and pushing them within safe boundaries. “*We try to have goals for each day, such as when we are visiting, she should say a word, or at school she should talk to at least one teacher. If she is going to buy something, she must ask for herself, and at soccer practice, she should say something to one of the adults*” (mother of a seven-year-old girl).

They emphasised that the goals had to be realistic. The parents tried to introduce new people and reassure their child in new settings by participating in leisure activities from the sidelines: “*We can contribute by encouraging her and continuously pushing her more and more to do things that she finds difficult and scary. We must not be afraid to challenge her. She will not be harmed by a little challenge. It is for her own good. The goals must be realistic and specific enough to be followed*” (parents of an eleven-year-old girl). The parents preferred to work “here-and-now” and observe the child’s reactions along the way rather than making a concrete progression plan with clear milestones at different times. Research shows, however, that working systematically and thinking long-term is appropriate with this issue (Omdal, 2007). The more days in silence, the more the child sees themselves as a person who does not speak and whom no one can help. SM becomes part of the child’s identity (Mead et al., 2015; Omdal, 2016). Two of the SM children started on Zoloft, an anxiety-reducing medication during the project, while three children were on medication at the onset of the intervention. It was unclear what the long-term effect of this would be. The father of a six-year-old thought it was good for his daughter that they did not talk to her about SM directly, in order to avoid her “becoming” her diagnosis such that the problem would be reinforced (Mead et al., 2015; Omdal, 2016).

#### 3.1.3. The Way Forward

The third step in Egan’s skilled helper model is about developing some action strategies that lead to constructive results. The goal in the parent group at this stage was to define concrete problem-solving actions from the prioritised goals. What should the parents do in order to make sure their wants and needs are met? Egan’s skilled helper model assumes active engagement from the parents, which not everyone had the capacity for. The parents expressed that coming up with solutions was the most challenging for them and something they needed considerable help with. They also tried to involve their children in discussions to figure out what could make it easier for them to talk and participate like other children: “*We are always talking about whether there is something we can do to help her at school or at home. Anything at all. But she does not have any answers herself*” (mother of a seven-year-old girl). “*To ensure that the goals are appropriately sized for her, we think it is important to listen to the child and observe her in the challenges she faces. If she becomes stuck or shows signs of discomfort, it is important to adjust the goals. If she finds the challenges too easy, it might be wise to increase the level of difficulty*” (parents of an eleven-year-old girl).

The parents in the group were helped to see that there were several ways to achieve goals. It is often necessary to specify the goals so that their meanings become clearer (Blase et al., 2012; Domitrovich et al., 2012; Durlak & DuPre, 2008). Clear goals facilitate the planning of concrete actions. This process needs to be given enough time, otherwise it becomes difficult to put the actions into practice. To achieve a sense of mastery, it is essential that each parent chooses a path that makes sense and aligns with their personal qualities, contextual factors, resources, style, temperament, and time frame (Egan, 2014). The parents received help in creating a systematic and targeted plan for the change process and assessed whether they seemed to be achieving their wants and needs. They received guidance on factors that facilitated or hindered the fulfilment of the plan and aspects they could continue to work on. Furthermore, they received feedback on their efforts and were encouraged to continue their change process.

According to the authoritative parenting style (Baumrind, 1991) the participants had been introduced to, parents are expected to take control of the situation and find the right balance between support and challenge in the relationship. In the following, parents reflect on their dilemma of providing their child with appropriate challenges: “*What we need to change about ourselves is that we need to let go a bit more of the idea of being worried about our daughter in tasks that she needs to practice. We need to dare to push her a little. What can hinder us from reaching our goals is largely ourselves. We need to try to set aside a couple of times a week to take her to different activities and exercises, such as shopping at the store, going to the swimming pool, and asking her teacher to ask her simple questions she can try to answer*” (mother of an eleven-year-old girl). “*It is a constant balancing act. You must pull back a little, but not too far. And you should challenge the child all the time, but not so much that it tips over, so it goes the wrong way again. It is difficult*” (mother of a seven-year-old girl).

A father in the group talked about his plans for his daughter’s progress as follows: “*What would be most beneficial is to encourage (but not pressure) her to seek out more settings where she can feel safe without us parents being present (for example, visiting friends and staying overnight with them). I need to try to help her become more independent in that regard. I should try to step back a bit when she is with others, as the impression is that it is easier for her to talk to new children (even adults) when I am not present and can ‘help’ her communicate. I should be more conscious about not answering for her when some adults ask her something and she does not respond or at least give her more time before I answer for her*” (father of a six-year-old girl).

Some parents were sceptical about exposing their daughter specifically to speaking: “*Activities that put her in the spotlight can quickly become negative. It might be better for her to be part of a group, so it does not seem so scary, and she does not feel like everyone is only looking at her. Challenges that solely involve talking to others can be intimidating*” (parents of an eleven-year-old girl).

A mother talked about how they exposed their son to speech using the mobile phone: “*We use video a lot. We actively use the phone in our daily life to get his voice going. We send a lot of videos to the grandparents. If I talk to an aunt or someone similar, I always put the phone on speaker, so he can chime in if he wants to. We also use Facetime quite a bit. He thinks it is great, loves to see himself. We use it in kindergarten as well. They get very emotional. They have never heard his voice. One staff member started crying, and then I started crying too, because it is so sad that they do not see him. It is like two different people. I really believe in this because he likes to be in the spotlight through the phone. I have asked him if I can show it to the other children in kindergarten, and he thinks that’s okay. Then the other children say: ‘There you are talking!’ and he stands there next to me smiling*” (mother of a six-year-old boy).

In some families, rewards were given for speaking outside the home: “*She counts the words she speaks because she is very eager to earn a prize. It started with 100, but then she wanted to increase it to 1000 to get a bigger prize. We had a reward where she could take all her friends she talks to to the cinema*” (mother of a seven-year-old girl). Humour and the opportunity to speak to people who had no expectation that the child would not speak seemed positive for some: “*What we can do more of that we notice has a positive effect on her is to use a lot of humour and joy. Her teacher also uses a lot of humour, and we see that it is positive for her. Another thing we have noticed is that she is not as afraid to talk to people who are not aware of her diagnosis. She has talked to several people who do not know that she is mute. This seems easier for her and is something we can work on further. Let her be in situations where there are people who do not know about her challenges and allow her to experience mastery by being able to talk to them*” (parents of an eleven-year-old girl).

In step 3 of Egan’s skilled helper model (2014), each family had to work out certain measures based on the goals they had formulated for their child in Egan’s step 2. The measures were based on the child’s challenges and possibilities for change. They should be in accordance with evidence-based practice following the authoritative parenting style (Baumrind, 1991). According to Egan (2014), the one who seeks advice must be active and take the responsibility for the change process in order to succeed. Implementation research supports this (Durlak, 2015). One must own the measures to get motivated to fulfill them. A facilitator cannot tell the other one exactly what to do. They know their own situation best. If it gets hard, the parents would blame the facilitator for not offering useful advice (Bandura, 1997). The measures operationalised from the core components of the intervention had to be specific and clearly formulated. The various measures must also be timed and responsibilities distributed within the child’s network. The implementation plan should also provide information on timing, content, and execution of regular evaluations. A mother highlighted the necessity of having small goals: “*It is important that the goals are not too high, but to rather have several small sub-goals that make it easier for her to feel a sense of mastery. Our goal is that she should be able to go to the store alone and buy something by herself. Other goals include feeling so secure at school that she can function normally there. We also aim for her to be able to talk normally with her grandparents. But we believe it is important not to do everything at once, but to take it step by step*” (parents of an eleven-year-old girl). A mother wanted to try inviting friends over: “*I talked to him about inviting one or two home. He agreed. Maybe someone he can play with. So, there is a small hope that it might get better at home*” (mother of a ten-year-old boy).

### 3.2. The Support System

Succeeding with an intervention depends on a strong support system (Blase et al., 2012; Domitrovich et al., 2012; Greenberg et al., 2005). The project leader tried to facilitate a common understanding in the parent group by having a shared framework for the group discussions using Egan’s skilled helper model (Egan, 2014). The participants also attended joint courses on the core components of the intervention, where they could bring key people from the child’s network. A couple of families brought grandparents, and one set of parents brought the child’s special educator. In addition, the parents had access to a research-based book in Norwegian about selective mutism as part of the support system (Omdal, 2016). They could approach the facilitator at any time with questions they had along the way, but few took advantage of this opportunity. Although the parents did not necessarily answer the reflection questions between assemblies, some still felt that these had made them think more closely about their situation: “*This has been useful for my own reflection. The systematic approach to this has forced you to think more closely about the details. It has been helpful, even though I have not had enough time to write anything down and do this in a way that is understandable to others besides ourselves*” (father of a nine-year-old girl).

### 3.3. Commitment to the Plan

There was a fair amount of variation in how committed the parents were to the different parts of the project, and what they said they would do did not always match what they conveyed they were doing together with the child and those around them. The number of participants varied at the gatherings, and the participants were generally poorly prepared for the questions based on Egan (2014) when they arrived. All nine families were present on the first assembly. Then there were four to five families present on the following assemblies through the year. Three families never came back. Only one family was always present as part of their agreement with the helping services. An excessive gap between plans made and action taken results in a weak implementation quality and little effect on the child (Blase et al., 2012; Domitrovich et al., 2012; Durlak, 2015). There were some negative expectations in the air concerning what parents thought the child would be able to master. Sensitive children will pick up on these expectations and live up to them (Mead et al., 2015; Omdal, 2016). The topic of expectations was addressed during the parent assemblies, so everyone should have been aware that this is a central core component of working with the child.

All the parents believed that safety was fundamental for helping the child to escape the silence. At the same time, several tried to be goal-oriented and challenge the child’s boundaries: “*For us, safety will be the most important thing. We believe that a basic sense of security can help reduce the gap between the current situation and the desired situation. Then, she must first and foremost feel safe at school and in other situations that may be scary for her*” (parents of an eleven-year-old girl). “*If she shows signs of a sense of mastery, we have succeeded with our goals and can move on. But if she shows signs of a lot of frustration and anxiety, we probably need to change the challenges and help her feel secure. If it should come to a halt and she regresses, we need to revisit our goals and see if there is anything that can be changed, and whether there is something we may have overlooked that could lead to a better outcome*” (parents of an eleven-year-old girl). Some participants were not as inclined to create goals and strategic plans to transition from the current situation to the desired situation according to Egan’s skilled helper model (2014). The following statement during the evaluation of the project seemed to indicate that some parents were placing responsibility outside themselves, or did not understand how to use the theory and be action-oriented: “*Difficult to answer questions like ‘How do you plan to achieve this?’ I do not know, that is why I am here*” (father of a nine-year-old girl).

### 3.4. Shared Visions

Through joint reflection, guidance, knowledge sharing, and training in the parent network, the goal was to expand the repertoire of everyone in handling selective mutism and to develop a common vision in the group. The shared learning was intended to enhance the overall knowledge and sense of mastery of both the individual and the group (Bandura, 1997; Leithwood, 2019; Leithwood & Beatty, 2008). Some highlighted in the evaluation that it became clear to them that the children and situations of the families were very different, but that they had received some input on what kind of help others were getting and what they could expect. One of the participants talked about how she had to independently seek knowledge about selective mutism when her child received the diagnosis: “*Before I came here, I had read everything there is about selective mutism and had studied this for three and a half years. If I had come here when I did not know what selective mutism was, I would have received those tips right away and would not have needed to spend three and a half years figuring everything out myself*” (mother of a seven-year-old girl).

It was intended that the participants would collaborate and motivate each other to sustain the process over time (Hargreaves & Fullan, 2012). The parents said that it was good for them to be in a group with other parents in the same situation: “*We did not know anyone beforehand and felt very alone. So, it is nice to talk to others who feel the same way and get confirmation that what we are doing is the same as what others are doing, and that it is good enough”* (mother of a ten-year-old girl). “*I have had a very strong need to exchange experiences and know that there are others in the same situation. It is good to share and see that I am not alone*” (mother of a seven-year-old girl). The supportive community in the group motivated them to revitalise the parents’ association for selective mutism in Norway as a continuation of this research project: “*I have gained a boost in my confidence that I am a good enough mom. I have struggled a lot with that. One becomes so disheartened. Just coming here, I just cried and cried the first time. It is so good! This is my biggest wish for everyone. We have googled ourselves to death on this topic. I wish everyone in my situation could be with someone who can say how they feel and know that it is perfectly fine. It gives you more confidence to dare to stand at that parent meeting and say it. To dare to go to soccer practice and reach out to the soccer coach and talk about it. And then you feel secure about it. It has been very good*” (mother of a six-year-old boy).

Several of the parents were frustrated that those around them did not spend enough time connecting with their child, either because they did not understand the problem or did not want to: “*If anyone is going to get our daughter to talk, then they will have to sit with her for four hours straight. No one is willing to do that except for the close ones. So, it feels a bit like it requires a lot, and then you withdraw*” (mother of a six-year-old girl).

The parents also talked a great deal about how they collaborated with the schools: “*We managed to drill the teacher into making sure that our daughter always knows what is going to happen. She needs predictability. In kindergarten, it was more like; what is happening now, you know. At school, they have been great at ensuring that everyone has someone to play with. Before each recess, they go through to check that everyone has someone to play with. It is not allowed to go outside until everyone has someone to play with*” (mother of a seven-year-old girl).

## 4. Discussion and Conclusions

In this section, the results are discussed according to the theoretical framework of the project.

### 4.1. Possibilities and Challenges Involved in Creating Parent Capacity in a Parent Guidance Group for Children with Selective Mutism

Implementing theories, structures, and activities in practice to reach goals is the most complicated and critical phase of an innovation to achieve change (Fullan, 2016). Therefore, in our project, the participants practiced implementing the theoretical principles they had read about in the book on selective mutism (Omdal, 2016) and learned about during the assemblies throughout the implementation year (Fixsen et al., 2005).

While the participants generally enjoyed the support of other parents in the group and not having to face the problem alone, they struggled with Egan’s (2014) third step, the action level. They found it difficult to implement the evidence-based principles of the innovation in their own situations. Implementation is about employing a theory or selected core component in concrete practical situations (Fullan, 2016). It is crucial that the theories are understood and a sound plan is developed for integrating the recommended principles into the family’s daily life in a way that will likely create lasting change (Meyers et al., 2012). If the implementation is not carried out well, or if there is a discrepancy between the planned innovation and actual actions, the effect on the child will be absent (Domitrovich et al., 2012; Greenberg et al., 2005). Loyalty to the principles of the innovation has the strongest impact on the quality of the implementation of the theories and the participants’ motivation to adhere to measures (Durlak & DuPre, 2008; Roland, 2015). There was considerable variation in the degree of commitment the parents had to the different parts of the project, and the action they said they would take did not always align with the action they reportedly were taking with the child and those around them. A significant gap between planned and actual actions results in weak implementation quality and little impact on the child (Blase et al., 2012; Domitrovich et al., 2012; Durlak, 2015).

These difficulties with taking action and implementing the innovation’s principles were also associated with certain negative expectations regarding the child’s potential mastery level. Some negative expectations were expressed, which sensitive children will pick up on and live up to (Mead et al., 2015; Omdal, 2016). This was the case even though the topic of expectations had been addressed during training and everyone should have been aware of this core component in working with the child. This may well have been a manifestation of exhausted parents wishing that others would solve their problems for them. They did not always believe that creating an implementation plan and specific goals for the child was a good idea, which made it difficult for the project leader to understand how they intended to take responsibility for creating change. After all, in addressing specific phobias, a successful outcome depends on systematic efforts where the child is gradually exposed to their fear (American Psychiatric Association [APA], 2022; Omdal & Galloway, 2008).

Those parents who had a child who was on medication had a boost in their motivation for making change happen. The child seemed to gradually relax and participate a little more when having Zoloft and started to say a few words to people they normally did not speak to. Still, the parents said that they had to continue working systematically with exposing the child to situations he or she perceived as frightening and adapt to the child’s special needs in various situations. Four of the families who believed in their own approach and implemented it over time in interactions with their child experienced that the child recovered from SM a couple of years after the project. Their strong sense of self-efficacy (Bandura, 1997) was crucial in their success. A sensitive SM child might copy parents’ positive beliefs in their strategies.

If a similar capacity-building project was to be carried out in the future, it would be beneficial to have fewer parents in the group and to ensure that the parents were committed to being involved in all parts of the project. Variable attendance complicated the project leader’s ability to move the project forward (Fixsen et al., 2005). It also complicated the group dynamics and the flow of the development process, demonstrating that loyalty to the initiative is a prerequisite for benefit (Greenberg et al., 2005; Hargreaves & Fullan, 2012). Furthermore, the parents had different capacity when it came to utilising the knowledge gained and following up on the guidance received. This, in addition to differing perceptions of their role as change agents in their child’s life, further stresses the benefit of having fewer participants.

### 4.2. Implications for Practice

In cases of selective mutism, capacity building is required to move from silence to speech. Parents are viewed as crucial change agents for the SM child. At the same time, without guidance from professionals with SM expertise, parents are also potential risk factors for the child’s progress (Omdal, 2014). Participants’ positive response to the programme, loyalty to the project, and a perceived need are prerequisites for achieving capacity building or an innovation, i.e., a planned change intended to improve practice (Greenberg et al., 2005; Hargreaves & Fullan, 2012; Skogen, 2004).

In our project, the main question was how to promote greater independence in parent–child relationships. Everyone felt the need for the child to begin speaking, and the families were generally concerned about finding the right balance between supporting and challenging the SM child in communication with others. However, some parents were perhaps not prepared for what this would involve on their part in terms of planned, targeted, and systematic efforts. One particular difficulty in this project was the apparent need to delve into individual family situations when challenges became significant. Additionally, some parents did not appear to see their own influence on their child’s behaviour. Everyone hoped that the focus would be on getting tips to help the children speak. From this perspective, perhaps it felt less useful to go through questions regarding thoughts about one’s own role.

Some important implications can be drawn from the project. The support system will benefit from closely and intensively following up on families with children who have selective mutism as soon as the condition is discovered. This is to avoid the communication pattern becoming locked over time. Parents can also benefit from having someone observe activities in and outside their home to identify potential maintaining factors (Nordahl & Hansen, 2012) that perpetuate the child’s silence and withdrawal. It is challenging for parents to view themselves and the child objectively, since they are an inexorable part of the relationship. And, as the years go by, the family may develop some unfortunate coping strategies that can hinder the child’s exploration of the outside world (Omdal, 2016).

For some parents, leaving their child at school and connecting their child with other children were challenging: “*During recess, it feels a bit strange for me to reach out to the other pupils. He says that if I leave, he will go with me. He is afraid of what the others might say to him. He feels that if I am there, they will not dare to say anything mean to him. ‘If you are not here, mom, then who am I supposed to talk to*?’” (mother of a ten-year-old boy). Many parents struggled to be in the child’s nearest zone of development while staying a step ahead in terms of expectations of what the child should attempt (Vygotskij et al., 1978). Some parents became an all-consuming supportive scaffold for the child, eliminating the child’s chance to communicate or act independently (Wood et al., 1976). Close monitoring over time is needed to try to help parents become authoritative adults who balance support and demands appropriately in their caregiving (Baumrind, 1991). It is difficult to see oneself from the outside and avoid projecting one’s own anxiety onto the child (Mead et al., 2015; Omdal, 2016). Having new eyes to analyse the parent–child relationship can be helpful if the goal is to change reactions towards the child.

Challenges both during and after the innovation added complexity and additional work at other levels. The longer the condition has lasted, the more complex the picture seems to be. More supervision after the end of the project year was needed both by parents and professionals in each case to help the children recover from SM. The fact that many of them were cautious people themselves or felt guilty that perhaps the child had ‘inherited’ their vulnerability may have led to them not being assertive enough to get the help they were entitled to. A mother talked about how she had to figure out her child’s rights: “*I went home and sat down to read the Education Act. Now I had to find out what she was entitled to. No one tells me. You must constantly learn about what exists and then try to get it. It is so hard*” (mother of a seven-year-old girl). The availability of a publicly approved parent association as a result of the research project could strengthen the parents’ hand, highlight children’s rights, and make their condition more understandable and better recognised in society.

Better verbal information about the project at the first assembly in addition to the written information they had received and signed, would probably have made the implementation easier. The researcher should have been clearer about her expectations and made the framework of the project clearer from the start. There were so many families eagerly telling their story at step 1 of Egan (2014) at the first meeting. It was overwhelming for the researcher to receive all that information from everyone at a time, trying to note as much as possible to save the participants’ background information for the further process. It would be recommended to involve another researcher into the project from the start. If it had to take place during the weekend, this was unlikely to happen. The role as both facilitator and researcher in the project was complex. The participants were asked to control the information noted in their case and read the interview transcripts to ensure that we got it right. One family added more information in their case. It helped a little to include a higher-level student assistant halfway into the project. The student assistant and the researcher reflected together on what happened during the assemblies. She transcribed the focus-group interviews and helped with practical issues during the assemblies. It was also a challenge when the participants varied from assembly to assembly that they had to update each other on information they had already given about their case previously. It delayed the implementation process and was ineffective for those who used to come. One could also question if Egan’s skilled helper model (2014) is too demanding for some parents who do not rely on their own decisions. Maybe the researcher thought that the parents were more independent than they appeared to be. The participants were informed that they could contact the researcher at any time during the process, but very few did. More frequent feedback during the implementation process would be appropriate in the most complex cases.

## Data Availability

Data is unavailable due to ethical restrictions (Selective mutism is a low-frequent group of children, and in a small country as Norway, we have to be cautious not to reveal the affected families. To protect the anonymity of the participants, the dataset is not available).

## Abbreviation

The following abbreviation is used in this manuscript:

SM	Selective Mutism
